# Feasibility of continuous smart health monitoring in pregnant population: A mixed-method approach

**DOI:** 10.1371/journal.pdig.0000517

**Published:** 2024-06-05

**Authors:** Zahra Sharifi-Heris, Michelle A. Fortier, Amir M. Rahmani, Hamid Sharifiheris, Miriam Bender

**Affiliations:** 1 Sue & Bill Gross School of Nursing, University of California, Irvine, California, United States of America; 2 UCLA School of Nursing, University of California, Los Angeles, California, United States of America; 3 Center on Stress & Health, University of California, Irvine, California, United States of America; 4 Department of Computer Science, University of California, Irvine, California, United States of America; 5 Azad University of Ahar, Department of Computer Science, Ahar, Iran; University of Turku: Turun Yliopisto, FINLAND

## Abstract

The utilization of smart monitoring technology offers potential for enhancing health outcomes, yet its feasibility and acceptance among Hispanic pregnant individuals remain uncertain. This is particularly crucial to investigate within the context of apparently healthy individuals identified as low risk, who still face a 10% likelihood of complications. Given their frequent underrepresentation in healthcare services and relative lack of attention, improving the feasibility of remote monitoring in this population could yield significant benefits. To address this gap, our study aimed to adapt and evaluate the practicality of a smart monitoring platform among healthy Hispanic pregnant women during the second and third trimesters of pregnancy, as well as one week following childbirth, a period when complications often arise. This longitudinal study followed n = 16 participants for an average of 17 weeks. Participants were instructed to wear the Oura ring for objective data collection, including activity, sleep, and heart rate, and to complete survey questions through REDcap to assess mental health and lifestyle factors. The study framework utilized the RE-AIM approach, with acceptability and adherence as key components of the feasibility evaluation. Our findings revealed that completion rates for biweekly and monthly surveys remained consistently high until after childbirth (approximately 80%), while daily question completion remained above 80% until 38^th^ week of gestation, declining thereafter. The wearing rate of the Oura ring remained consistently above 80% until the 35th gestational week, decreasing to around 31% postpartum. Participants cited barriers to wearing the ring during the postpartum period, including difficulties managing the newborn, forgetfulness, and concerns about scratching the baby’s skin. The enrollment rate was 71.42%, with an attrition rate of 6.25%. Thematic analysis of one-on-one interviews identified three main themes: personal desire for health improvement, social acceptability and support, and conditions influencing device/platform efficiency. In conclusion, while adherence varied based on gestational week and survey frequency, the study demonstrated strong acceptability of the smart monitoring platform among the study population, indicated by the high enrollment rate. Qualitative insights underscored the significance of personal motivation, social support, and device/platform efficiency in enhancing patient engagement with digital health monitoring during pregnancy, offering valuable considerations for future healthcare interventions in this domain.

## Author summary

Our team is committed to advancing the understanding of smart monitoring technology’s feasibility and acceptance among Hispanic pregnant individuals. This research is imperative given the disparities in healthcare access and attention faced by this demographic. By conducting a longitudinal study, we aimed to assess the practicality of a smart monitoring platform throughout pregnancy and postpartum. Our findings highlight the importance of addressing barriers to adherence and the significance of personal motivation and social support in promoting engagement with digital health monitoring tools. This study emphasizes the potential of smart monitoring technology to improve healthcare outcomes in underserved populations and provides valuable insights for designing future interventions. As advocates for equitable healthcare access, we are dedicated to translating these findings into actionable strategies to better support the health and well-being of pregnant individuals in Hispanic communities.

## Introduction

Traditional face-to-face health assessments are increasingly being augmented by remote and intelligent monitoring technologies, presenting significant advantages for both healthcare providers and patients. The adoption of remote monitoring has been linked to a variety of health benefits such as decreased hospital admissions, shorter stays, reduced need for emergency procedures, better health outcomes, alleviated strain on healthcare infrastructure, improved chronic disease management, lower risk of infectious disease transmission, and savings in both time and costs associated with healthcare procedures [1–2]. Today, with many of the previous restrictions lifted following the Covid-19 pandemic, there is heightened recognition and potential for application of remote monitoring in enhancing healthcare delivery systems.

Research indicates the significant outcomes of incorporating smart wearable devices in prenatal care including, health and safety monitoring, habit improvement (e.g., sleep, activity, nutrition, self-awareness), chronic disease management, diagnosis, and treatment (cardiovascular complications, hypertensive disorders, diabetes, pulmonary disease) [3–7]. The smart wearable devices, capable of capturing pertinent biomarkers continuously, address barriers to disease identification and treatment while facilitating disease prediction by providing continuous data. For instance, heart rate variability (HRV) has been suggested as a biomarker for hypertensive disorders in pregnancy [8–9] When assessed continuously, HRV shows promise in the early identification of hypertensive disorders as an important leading cause of maternal mortality [10]. By enabling monitoring of lifestyle factors such as activity and nutrition, smart wearables also hold potential for improving pregnancy outcomes [11–12].

In this context, to leverage resources for integrating smart monitoring, assessing feasibility of smart monitoring systems in prenatal care, especially among underserved ethnic minority individuals. Existing screening protocols may not adequately address the needs of ethnic minority populations, necessitating a closer examination of how smart monitoring can bridge gaps in healthcare access and outcomes. This assessment is particularly crucial for healthy-identified pregnancies, where complications may still arise by 10% without prior detection [13–16]

Through feasibility assessment, we can pinpoint potential barriers and challenges that may hinder the smooth integration of these systems into clinical practice. Engaging stakeholders in this process allows us to grasp the various factors influencing the reach of these systems, particularly among vulnerable populations like ethnic minorities with higher risks of pregnancy complications.

Moreover, by identifying adoption barriers such as device usability issues or participant demographics, we can tailor our implementation strategies, accordingly, maximizing the adoption of smart monitoring systems. These insights not only help us overcome identified obstacles but also pave the way for sustained implementation of these systems, ensuring their long-term effectiveness in improving health outcomes for pregnant individuals.

While there have been studies conducted on the feasibility barriers to implementing smart monitoring in non-Hispanic pregnant populations, the feasibility of using smart platforms in healthy Hispanic pregnant individuals remains less understood. To bridge this knowledge gap, our study aims to tailor the feasibility of smart monitoring platform using evidence-informed strategies in healthy Hispanic pregnant women during the second and third trimester of pregnancy and one week after childbirth. This assessment, when guided with reliable implementation frameworks such as the RE-AIM framework, provides valuable insights that inform different aspects of implementation efforts.

## Materials and methods

### Study framework

The RE-AIM framework, established by Glasgow et al. in 1999 [17], comprises five domains: reach, effectiveness, adoption, implementation, and maintenance. It aids in translating scientific advancements into practical applications and addresses the challenge of implementing health protocols across diverse populations. By balancing internal and external validity, RE-AIM ensures interventions maintain efficacy in real-world settings. It emphasizes problem-solving and considers various factors in intervention design, dissemination, and implementation for broad population impact. The framework encourages protocol modifications to suit different populations, enhancing effectiveness. In the context of smart monitoring platforms, understanding user characteristics, context, and culture is vital. The framework guides the study’s conceptualization, evaluating components crucial for implementation success. "Reach" assesses accessibility and appeal, "Adoption" evaluates administrative readiness, and "Implementation" addresses needs and barriers. Effective implementation ensures "Effectiveness" and "Maintenance" for improved health outcomes. To conceptually understand the application of the RE-AIM components in assessing the feasibility of the smart monitoring platform we considered “adherence” and “acceptability” as two main components.

Adherence refers to the extent to which individuals follow the prescribed protocol or use the smart monitoring system as intended. This aspect ensures that the intervention is being accepted and followed effectively and that participants are engaged in the process. Acceptability, on the other hand, pertains to the willingness and satisfaction of individuals with the smart monitoring platform. It assesses whether the intervention is perceived favorably by users and whether it aligns with their preferences, needs, and cultural context. Additionally, it identifies any challenges encountered that may influence users to either persist with or discontinue using the device and platform. By considering adherence and acceptability within the RE-AIM framework, the study can gain insights into how well the intended intervention is received and utilized by the target population, what are the potential barriers, and how to overcome the proposed obstacles. The developed implementation strategies feed toward implementation success and ultimately can enhance the effectiveness and sustainability of the smart monitoring platform, leading to improved health outcomes for the target population.

### Study design

The study utilized a prospective longitudinal observational study design, incorporating a mixed-method (quantitative-qualitative) approach for data collection, analysis, and synthesis. The study commenced during the 22^nd^ gestational week and continued until one week after childbirth, covering a period of seventeen weeks on average.

### Sample and setting

Our study focused on pregnant individuals residing in the southern California region. To ensure ethical considerations, we obtained approval from the Institutional Review Board (IRB) before initiating the research. Participants were recruited by our researcher (ZS) from Manchester Clinic using purposeful sampling, and specific criteria were established to address the research questions. The inclusion criteria were as follows: 1) Participants needed to meet the health standards outlined by the American College of Obstetricians and Gynecologists (ACOG) (see S1 Appendix for details), 2) Gestational age between 20 and 24 weeks, 3) Participants needed to identify as Latina ethnicity, 4) Proficiency in the English language was required, and 5) Participants needed to have access to the internet and possess a smartphone.

Electronic Privacy Information Center (Epic) was monitored biweekly for the assessment of both inclusion (recruitment phase) and exclusion criteria (during the entire study course) to assure healthy status of the pregnant participants. Additionally, REDcap was consistently monitored for the potential self-reported mental and/or physical health conditions verifying eligibility criteria throughout the study duration.

### Sample size

Since this study is a pilot study without existing information to guide the sample size determination, the literature suggests a sample size of twelve for preliminary data collection and analysis [18]. Recent statistics indicate that around 10% of low-risk pregnancies have the potential to develop complications [16] Accounting for a potential attrition rate of 15%, the final sample size was determined to be n = 16.

### Assessments and measures

The conceptualized feasibility of adherence and acceptability in individuals toward the use of Oura ring and Research Electronic Data Capture (REDcap) platforms were assessed as the main measures of the study. Video-conferencing was leveraged as the modality to interact with the participants during the one-on-one interview to qualitatively assess their personal experiences toward using the smart platforms of Oura and REDcap surveys.

**Oura ring:** Oura ring was used to continuously monitor lifestyle-related factors including activity, sleep, heart rate, and physiological biomarkers. Oura has received FDA clearance and European Conformity (CE) marking through Natural Cycles (NC), a cycle tracking app. It is a waterproof smart ring crafted from scratch-resistant Zirconia, equipped with sensors like PPG, 3D accelerometer, Gyroscope, and NTC body temperature sensor. Oura has been utilized and validated in prior research for its various features including activity, sleep, heart rate, heart rate variability, and energy expenditure [19–24]. The study participants were encouraged to wear the ring as frequently as possible during the course of study and report any incident or issue hindering them from wearing and maintaining the ring.

**REDcap:** REDcap platform was applied to subjectively assess mental distress (stress, anxiety, depression) and lifestyle factors using survey questions. As a secure web application, REDCap was chosen for constructing and managing our online surveys securely. Participants received scheduled survey questionnaires as email links and were encouraged to complete them to the best of their ability. Mental distress assessed using three validated scales of PHQ-2 for depression, PSS-4 for stress, and GAD-2 for anxiety, all three have demonstrated reliability and validity in pregnant individuals with various education levels in both urban and rural settings [25,26]. They use a scale with two or four items to measure symptoms over specific time frames (two weeks for PHQ-2 and GAD-2, and one month for PSS-4). Higher scores indicate more severe symptoms.

We employed a daily survey questionnaire to subjectively evaluate lifestyle-related factors such as activity, sleep, stress, and diet, as reported by the participants. This complemented the objective assessments provided by the Oura ring. The questions were distributed at the end of each day, before the participants went to bed.

**Video-conferencing:** The participants were interviewed for eight biweekly sessions via the Zoom platform. To assure the credibility and reliability of the findings, a triangulation approach was employed, leveraging multiple encrypted data recording sources including anonymous voice-recording and detailed note-taking. While participants led the interview, some researcher-developed questions were prepared to maintain focus on the main research aim. These questions included:

1. How often do you wear the ring?
2. How do you feel about wearing the ring?
3. Any negative and positive points since our last meeting?
4. How do you feel completing the survey questions?
5. Are survey questions helpful or painful when completing?
6. Do the survey questions conflict with your daily life?

The audio recordings were transcribed into a written catalogue enriched by the notebook data. To ensure the consistency and accuracy of the interview data, the audio tapes were listened for several times in different time periods and transcribed accordingly. During the transcription process, we contacted participants for any vague responses or those that required further explanation or clarification. From the transcribed files, the excerpts were organized in the excel sheet for the consequent code and theme extraction.

### Study procedure

After obtaining approval from the Institutional Review Board (IRB), we utilized purposive sampling to identify potential candidates from the designated clinic. Access to the EPIC system (electronic health record) in the clinic, granted by the IRB, allowed our researcher (ZS) to pre-identify potential participants based on the study’s predefined inclusion criteria. Prospective participants meeting the criteria were contacted during their prenatal appointment if they still met the required gestational week at the time of appointment. Before the visit, the assigned OB/GYN care-provider was informed about the individual’s potential participation. The OB/GYN took the first step introducing the study during the visit, and if initial interest was indicated, the individual was referred to ZS for further eligibility assessment and potential recruitment. Details of the study were provided to interested and eligible individuals, and consent forms were obtained if still showed willingness for participation. Participants who completed the consent process proceeded to the monitoring phase (entire study course after recruitment), during which study measures were being monitored at the specified frequencies outlined in the "Assessments and Measures" section: Oura ring continuously; REDcap (mental surveys depending on the scale biweekly or monthly); Zoom interviews biweekly. In case of less than 30% weekly wear-time for Oura or failure in competing more than one survey sessions participants were connected for potential support addressing the existing technical and/or non-technical barriers. To assure eligibility criteria across the study, Epic and REDcap were monitored consistently. Participants who showed no complications or risk factors by the end of the study course proceeded to the analysis phase. Those with identified complications excluded from the study and referred to appropriate healthcare providers for potential interventions.

### Sampling

**Fig 1:**
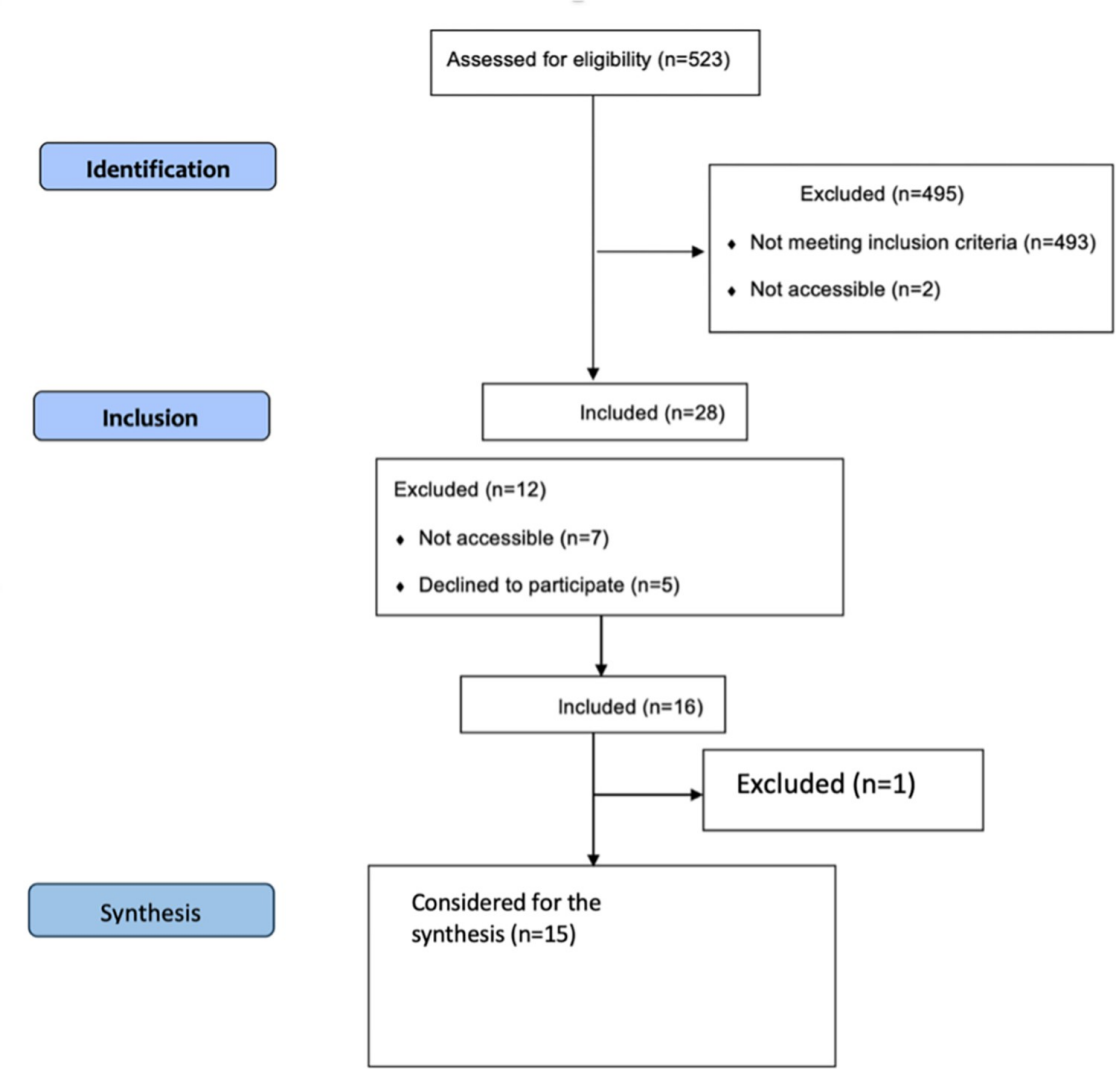
STROBE chart.

The study recruited participants from a Manchester clinic associated with UCI, starting from August 21^st^ and continuing until November 16^th^, 2022, with the aim of enrolling a total of 16 individuals who met the inclusion criteria. Initially, 523 individuals were assessed for potential eligibility using the EPIC system. Among them, 493 did not meet the inclusion criteria, and 2 were excluded as they were receiving prenatal services elsewhere. Ultimately, 28 individuals were identified as eligible for the study. However, challenges in the recruitment process emerged. Seven participants did not attend their scheduled prenatal care appointments, and five declined to participate in the study. Among those who declined, three required their husband’s permission, while two provided no specific reason. Eventually, 16 participants were successfully enrolled in the study. During the course of the study, one participant had to discontinue due to a hand injury, preventing them from wearing jewelry as prescribed by their care provider. Additionally, another participant experienced a complicated pregnancy (placental abruption) at 34 weeks and was subsequently excluded. As a result, a total of 14 healthy participants and one complicated case were considered for the final analysis and synthesis. It was decided to include the complicated case since her perspective when she was healthy (right before 34^th^ gestational week) is still valuable and representative of healthy pregnant individuals’ viewpoints (See Fig 1).

## Data analysis and synthesis

The study employed a qualitative-qualitative research design to thoroughly analyze and synthesize the data, integrating both deductive and inductive approaches to explore the complexity of the phenomenon under investigation [27].

### Quantitative

#### Adherence

The adherence was assessed for both the Oura ring and the REDcap app. We monitored for potential barriers experienced by the study participants during the study (Reach). Adherence to wearing the Oura ring in a given week is determined by calculating the proportion of the recorded data duration (regardless of its quality) to the total expected duration for that week (24 hours x 7 days = 168 hours). For instance, if a participant wears the ring for 125 hours in a week, the adherence percentage would be approximately 74% (125 hours / 168 hours).

For the survey questions on the REDcap platform, on the other hand, the response rate for “daily questions” was the proportion of number of daily surveys completed over the week to seven (number of days that participants were supposed to complete the surveys). For the biweekly and monthly surveys, if the participant completed the biweekly survey, the rate was 100% and if not 0 in the relevant time period.

Please note that each survey can only be completed once by the participants to eliminate the risk of duplication error.

#### Acceptability

For acceptability assessment, we used enrollment and attrition rate to assess acceptability and to assess how implementation strategies developed through the feasibility assessment are effective in minimizing the attrition rate. The enrollment rate is the proportion of the enrolled individuals to those who are invited. The attrition rate is the proportion of excluded individuals during the study to those who were enrolled in the study.

Administrative barriers in technical aspects, maintenance, and instruction were obtained in as an incidence report by participants and technical support people in our research team. If a participant’s record in the week was showing an abnormal data or shortage of data recorded by ring (less than 30% wear-time for the ring and more than one missing survey), we proactively contacted the participant for potential technical difficulties or personal concerns that might be the hindering factor.

### Qualitative

#### Personal perspectives affecting adherence and acceptability

A relational content analysis (deductive-inductive approach) [28] was considered to understand the participants’ experiences and toward application of smart platforms for health monitoring. This approach enables us to explore the relationships between variables while also testing theoretical research assumptions. Indeed, it combines the flexibility of inductive analysis with the rigor of deductive analysis to obtain a deeper understanding of complex relationships between different concepts in the captured data.

We first underwent inductive analysis to minimize the bias and reduction in the deductive approach. For this, the data were exported to an excel spreadsheet, where inductive coding began considering all possible concepts extractable from the interview data in the current study including those that may or may not exist in the existing literature. Then, we conducted deductive coding, comparing the codes and themes extracted from our study with the ones in the existing literature to understand how our excerpts may or may not confirm or conflict with the existing concepts regarding the research topic.

## Results

### Descriptive information

Upon enrollment, our study included 15 participants whose descriptive characteristics were examined as indicated in Table 1. The mean age of the participants was 31.7 years all either equal or under 35 years old. On average, the mean BMI was 24.41, all in healthy range (18.5–24.9). Their total educational attainment averaged 18.24 years. Regarding gravidity, 67% (10/15) of participants had undergone two pregnancies, while the rest had experienced either three or four pregnancies. About 67% (10/15) had one childbirth, and the rest either two or three childbirths.

**Table 1:** Descriptive characteristics.

Characteristics		Findings
**Age (years) (mean [SD])**		31.7 (3.89)
**BMI (mean [SD])**		24.41 (2.23)
**Education (years) (mean [SD])**		18.24 (5.49)
**Income ($) (number [%])**	**<$100k**	13 (87%)
	**≥$100k**	2 (13%)
**Gravidity (number [%])**	**2**	10 (67%)
	**>2**	5 (33%)
**Parity (number [%])**	**1**	10 (67%)
	**>1**	5 (33%)

### Feasibility

#### Quantitative

Adherence

The adherence was assessed for both the Oura ring (wearing time) and the REDcap surveys (completion rate).

**Fig 2:**
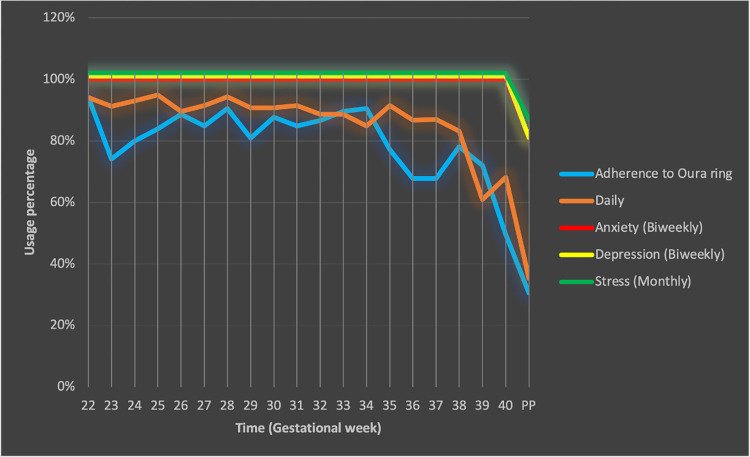
Completion rate of survey and wearing rate of Oura ring.

As shown in the Table 2 and the Fig 2, the completion rate of the biweekly (anxiety and depression) and monthly (stress) surveys were 100% until after childbirth where the rate dropped to around 80%. The completion rate for daily questions stayed above 80% until 38 weeks of gestation. Then it gradually decreased toward post-partum.

**Table 2:** Percentage of usage for Oura ring and survey completion.

	Adherence to Oura ring	Adherence to REDcap (survey questions)			
		Daily (Lifestyle)	Biweekly		Monthly (Stress)
			Anxiety	Depression	
Week 22 (n = 1)	94%	94%	100%	100%	100%
Week 23 (n = 4)	74%	91.23%			
Week 24 (n = 11)	80%	93%	100%	100%	
Week 25 (n = 15)	83.81%	94.90%			
Week 26 (n = 15)	88.57%	89.52%	100%	100%	100%
Week 27 (n = 15)	84.76%	91.43%			
Week 28 (n = 15)	90.48%	94.28%	100%	100%	
Week 29 (n = 15)	80.95%	90.74%			
Week 30 (n = 15)	87.62%	90.74%	100%	100%	100%
Week 31 (n = 15)	84.76%	91.43%			
Week 32 (n = 15)	86.62%	88.57%	100%	100%	
Week 33 (n = 15)	89.52%	88.57%			
Week 34 (n = 15)	90.47%	84.76%	100%	100%	100%
Week 35 (n = 15)	77.14%	91.43%			
Week 36 (n = 15)	67.78%	86.66%	100%	100%	
Week 37 (n = 15)	67.72%	86.93%			
Week 38 (n = 15)	78.09%	83.12%	100%	100%	100%
Week 39 (n = 13)	71.98%	60.95%			
Week 40 (n = 6)	49.52%	68.09%	100%	100%	
Week 41 (n = 1)	100%	50%			
Post-partum (n = 15)	30.47%	35.22%	81%	81%	87%

Oura ring wearing rate was above 80% from the beginning of the study until 35^th^ gestational week except for gestational week of 23 when the rate dropped to 74% across the participants. The wearing time after 35^th^ gestational week, more or less, tend to decrease toward postpartum when the wearing rate was about 31% across all the participants.

Postpartum poses the lowest average value for survey completion rate and wearing time across the participants.

Daily question completion significantly changed from week 38 to 39 (p<0.01) and from week 40 to post-partum (p<0.01). The wear time duration changes from gestational week of 22 to 23 (p<0.01), from week 34 to 35 (p<0.05), from 39 to 40 (p<0.01), and from 40 to post-partum (p<0.01) were statistically significant. None of the sociodemographic factors significantly affected the wearing time and daily survey completion over the period of the study.

The wear time in the one-week postpartum was lower than that during the rest of the study (*p* < 0.001). About 50% (n = 7/15) reported difficulty in managing the newborn while using the ring, 30% (n = 5/15) forgetfulness, and 30% concern to scratch the newborn’s skin as the excising barriers for wearing the ring during postpartum period.

None of the participants brought up the concern with charging and synching the device. They reported one-time full charge enough for about three days of full use. About 30% (n = 5/15) of the participants sometimes experiences lower than 30% of wearing time in some gestational weeks. Forgetfulness and busy schedule were reported inconvenience while handling the newborn, concern for harming the newborn, and forgetfulness as the possible barriers for the ad and were worried about whether the devices might scratch their babies. as the underlying reasons for this rate. Weekdays and weekend exposed no impact on the wearing time. About 70% (n = 10/15) of the participants did not were the ring during the childbirth.

Acceptability

We used enrollment and attrition rate to assess acceptability. The enrollment rate was measured as the proportion of the enrolled individuals to those who were invited. The attrition rate was the proportion of excluded individuals during the study to those who were enrolled in the study.

Enrollment rate:

$$X=\frac{15}{21}\;(100)=71.42\%$$

Attrition rate:

$$X=\frac{1}{16}\;(100)=6.25\%$$

#### Qualitative

Personal perspectives

During the period of participation, a one-on-one interview was conducted biweekly for a total of eight sessions (16 weeks). For the final three sessions (sessions 6–8), over 60% of participants reported difficulty in setting a time for one-on-one interview and showed desire for switching to a written format of addressing the interview questions as they did not have any new comments or concerns. As a result, we conducted the last three sessions by sending participants a set of questions via email and having them complete the questions in the written format. The interview took an average of 20 minutes to complete.

No technical issues were noticed for the under adherence under 30%. Participants (n = 5) reported forgetfulness (n = 2/5 40%—n = 3/5 60%) or busy schedule (n = 1/5 20%—n = 3/5 60%) as the underlying reasons for the low rate.

Emergent Codes and Themes

In *inductive approach*, after importing the data into an excel sheet, we began the coding process by reviewing each excerpt thoroughly and identifying key concepts and patterns with an attempt to decrease the potential bias. Using these insights, we created a set of emergent codes that captured the most salient themes in the data. From there, we synthesized these codes into overarching themes that provided a comprehensive understanding of the data as a whole. This process enabled us to identify patterns, relationships, and insights that would have otherwise remained hidden in the raw data.

In *deductive approach*, on the other hand, we provided preexisting themes in the evidence and purposefully tried to discover their potential link and relevancy to the themes extracted form inductive approach. To eliminate potential bias, we went over the excerpts for finding the hidden themes that could have a link with the preexisting themes and might have been undiscovered.

Conduction of inductive prior to deductive approach in the relational analysis enabled us to decrease the bias and reduction that otherwise might had led to the hidden and undiscovered concepts and themes.

Seeking relationships and clustering themes in deductive approach

During this stage of our research/analysis, our aim was to identify relationships between emerging themes and group them based on their conceptual similarities. By clustering the themes together, we were able to provide each cluster with a descriptive label. Please see the Table 3.

This approach helped us to gain a deeper understanding of the connections and patterns between the different themes, which in turn allowed us to draw more meaningful conclusions from our research/analysis.

**Table 3:** Theme clustering in inductive approach.

Codes	Themes	Subtheme/concepts
**Appearance**	Social Acceptancy and Desire	◊ Social support ◊ Social stigma ◊ Appearance
**Acceptance by others**		
**Mindfulness**	Support System for Health Awareness	◊ Real-time awareness and empowerment ◊ Reminding potential needs
**Value**	Personal desire and Value	◊ Technology application in health improvement
**Comfort**	Conditions Associated with the Device/Platform Efficiency	◊ Comfortable to use ◊ Accurate information ◊ Cost-effective alternative for patient-caregiver communication and maintenance ◊ Time-saving alternative for patient-caregiver communication ◊ Personalized accommodations in the platform ◊ Facilitating real-time access to the required information
**Validity**		
**Meeting immediate needs**		
**Accuracy**		
**Personalization**		
**Maintenance**		
**Time efficiency**		
**Straightforward**		
**Notifications**		
**Real-time info**		
**Physical accommodations**		
**Forgetfulness**		

The main themes categorized into four categories of personal desire, social acceptancy and desire, support system for health self-awareness, and conditions associated with the device/platform efficiency.

For deductive approach, the preexisting themes were from the existing literature that were concerned with qualitative assessment of patient experience in using smart platform for health tracking [29,30]. As shown in the Table 4, all preexisting themes were detected in our qualitative data except for “social acceptancy and desire”. For the other themes, the subthemes such as “reminding potential needs” and “personalized accommodation in the platform” were not identified in the preexisting subthemes. On the other hand, in our data we could not confirm two subthemes including “Data protection” and “Stakeholder-associated financial interests” in the preexisting data.

**Table 4:** Deductive-inductive approach.

*Inductive analysis*		*Deductive analysis*
*Themes*	*Subthemes*	*Pre-existing Themes*
Social Acceptancy and Desire	Social support	N.A.
	Social stigma	N.A.
	Appearance	N.A.
Support System for Health Awareness	Real-time awareness and empowerment	Empowerment and education Better awareness of personal medical history and body functioning Supporting patients’ self-diagnosis and self-medication
	Reminding potential needs	N.A.
Personal desire and Value	Technology application in health improvement	Behavior change facilitation
Conditions Associated with the Device/Platform Efficiency	Comfortable to use	Ease of use and design features
	Accurate information	Scientific validation
	Cost-effective alternative for patient-caregiver communication and maintenance	Efficient information transmission Connection with the patient’s medical network
	Time-saving alternative for patient-caregiver communication	Efficient information transmission Connection with the patient’s medical network
	Personalized accommodations in the platform	N.A.
	Facilitating real-time access to the required information	Better awareness of personal medical history and body functioning
N.A.	N.A.	Data protection
N.A.	N.A.	Stakeholder-associated financial interests

Personal Desire and Value

About 80% (n = 12/15) referred to their willingness in using the Oura ring and 66% (n = 10/15) gave appreciation in engaging with the survey questions sent through REDcap. The value was reflected in concepts such as being nice (75% (n = 9/12)), valuable/worthy (16% (n = 2/12)), and phenomenon in (8% (n = 1/12)) for the Oura ring and app, and as being nice (70% (n = 7/10)) and being desirable (30% (n = 3/10)) for survey questions that were sent through REDcap.

*I.* Technology application in health improvement is the main emerging subtheme for the “personal desire and value’ across the excerpts.

Participant 1: “*It is nice having it [Oura ring] on*. *I am impressed by how it reads my heart rate right away specifically when I work out*.*”*

Participant 6: *“The survey questions are nice reminders on how I have eaten and been acting throughout the day*.*”*

Participant 7: *“I have been told by previous doctors (pre-pregnancy) that my heart rate is sometimes on the higher end*, *so it is nice to be able to track that (heart rate) with this ring*.*”*

Social Acceptancy and Desire

About 86% (n = 13/15) of the participants pointed out the importance of how the device/platform use may affect others (e.g., significant other, family, and friends) perspectives toward them wearing the ring and completing the survey questions. This desire to social acceptancy was subcategorized into three subthemes of social support, social stigma, and appearance.

*I.* Social support: About 46% (n = 6/13) of the participants highlighted the significance of their surrounding people in interpretating their behaviors and consequent support or lack of support.

Participant 3: *“I will try to stay on the study unless my family… like their situation… change my mind*.*” “… what if my husband feels it is unnecessary…”*

Participant 5: *“Anyway I need his [her husband] support for my pregnancy and afterward to grow up my baby*, *so I need his thoughts as well whether it is fine to keep this [Oura ring] on*.*”*

*II.* Social Stigma: About 38% (n = 5/13) participants seemed concerned about how others may evaluate and judge their use of smart device or survey question completion.

Participant 9: *“I would like the survey questions to come through later in the day when I’m about to go to bed*. *I feel like this is the best time when no one is around” “It is not just distraction but actually*… *you know I’m not comfortable these texts come up and being seen by my colleagues or friends*.*”*

Participant 7: *“I’ve never seen like this… It may look like a digital jail that tracks everything… [laughing]*.*”*

*III.* Appearance: All the participants pointed out the importance of the way the ring looks to others representing their style or look in the society.

Participant 1: *“Is this the same thing used by Kim Kardashian*? *I would love to try this on as it is now a trend*.*”*

Participant 9: *“Sometimes people say that it is bulky and looks like men’s wedding ring*. *And specifically*, *some women think that it is blended with their other jewelry*. *Maybe making it more delicate can satisfy women’s needs*.*”*

Participant 3: *“I hope it is not going to ruin my style [laughing]” “It may fit with some of my clothings though*.*”*

Conditions Associated with the Device/Platform Efficiency

All participants indicated certain conditions that affect their adherence to the device/platform use. These conditions individually and/or in combination with each other may impact one’s desire.

I. Comfortable to use: All participants touched upon the importance of being comfortable when using the device/platform. They often used terms such as “easy” and “straightforward” for presenting their comfort in the excerpts.

Participant 2: *“I do not really feel the ring on*, *so no distraction*.*”*

Participant 7: *“I wear it (ring) all the time except for the shower and charging period and I don’t even feel it on*. *I also like its easy app to navigate through*.*”*

Participant 3: *“It’s just like a second to complete survey questions and it is not a big deal…*..*is pretty straightforward*.*”*

II. Accurate information: About 70% (n = 11/15) participants pointed out the matter of the device accuracy in reflecting the data.

Participant 7: *“There are lots of smart devices such as watches*, *braces*, *rings that are being used to track body*. *But this one feel fairly the most accurate one*.*”*

Participant 4: *“I was initially concerned about its [Oura ring] accuracy and how reliable is the reported data*. *But I am impressed how there are growing evidence proving its high accuracy…”*

III. Cost-effective alternative for patient-caregiver communication and maintenance: About 26% (n = 4/15) participants reflected the significance of the maintenance cost of the ring and 40% (n = 6/15) pointed out the importance of saving costs as a result of being monitored remotely for the health-related metrics as compared to in-person medical appoitments.

Participant 7: *“Due to a fluctuation in my heart rate*, *I’ve been suggested to be monitored regularly for my heart rate*. *But you know it is costly and time consuming to get here [clinic] just for getting checked for the heart rate*. *It is nice and cost-effective to track my heart rate with this (ring)*.*”*

Participant 3: *“I charge it every 4–5 days and it keeps the battery on very well*. *So*, *it is awesome*.*”*

IV. Time-saving alternative for patient-caregiver communication: About 20% (n = 3/15) of participants suggested the smart remote monitoring as alternative time-saving approaches for in-person appointment with their care providers.

Participant 11: *“I guess all is good about the ring and the app*. *It could be even better if we could communicate this info with the doctors*. *So*, *it would hopefully lower unnecessary time-consuming appointments*. *It actually serves telemedicine*. *Instead of spending hours traveling to and from doctors’ offices*, *we can now have virtual consultations from home*. *It saves me sometimes and money and also keeps me away from the stress in the clinic*.*”*

V. Personalized accommodations in the platform: All 15 participants referred to the importance of personalizing the device features and relevant interpretation of the app and survey questions based on the user’s context. For example, pregnancy normally accompanied with various physical and emotional changes. These pregnancy-induced expected modifications needed to be reflected in the data to hinder the misinterpretation of these modifications as pathological changes.

Participant 7: *“I guess one of the things they [Oura company] may consider is the customization of the notifications based on one’s lifestyle*, *characteristics*, *and mandatory life requirements*. *For example*, *for some people 5-hour sleep sounds enough and for some it is like nothing*. *Also*, *even if we need more sleep*, *our work schedule won’t allow us*. *Most of us are not able to change our work schedule and lifestyle*. *So*, *sending notifications to sleep more makes us even more stressed out*.*”*

Participant 3: *“My sleep length has gotten short for a few weeks now*. *But I do not feel tired or restless at all*. *I guess this is just the nature of pregnancy and is not a thing to worry about*. *Although the app is keep saying I need to take more sleep*, *I guess it just assuming a non-pregnant person*. *If it was my first pregnancy I would have stressed out*. *But since I felt the same problem in my previous pregnancy*, *I know it is normal*.*”*

Participant 4: *“I am on my feet all the time*. *I think depending on the individual*, *ring can provide helpful info*. *For me heart rate and activity aspect are the most important ones*. *I would like to see them in the home page of the app without going through further steps to figuring them out*.*”*

Participant 8: “I feel nervous as I am getting closer to the date [childbirth]. I think it is because motherhood brings lots of responsibility and stress in fact makes me to get up and do the required accommodations…. But not sure how scientifically this stress may impact my baby…. Is it normal or worrisome!”

VI. Facilitating real-time access to the required information: About 60% (n = 9/15) of the participants reported the benefit of having a real-time access to the data reported by the Oura app.

Participant 4: *“I like having a real-time access to my heart rate as it informs me the way my heart responds over the different phases of my exercise right away*.*”*

Participant 5: “It [Oura] makes me more knowledgeable and confident about what my body has been through. The other day I was experiencing contractions and my doctor asked about my activity level that might be the cause. I confidently looked at the data and provided accurate activity intensity to her addressing underlying cause for the contractions.”

Support System for Health Self-Awareness

About 60% (n = 9/15) of the percipients pointed out the matter of mindfulness as a result of using the ring and survey questions.

I. Real-time awareness and empowerment: About 60% (n = 9/15) of the participants reported their satisfaction for having a real-time access to the data that inform and empower them about their health situations and the rest (40%) were neutral in this regard.

Participant 7: “*Survey questions make me mindful and aware of what I have been through emotionally and what I’ve been eating all day*.*”*

Participant 11. *“You know how your body act when you do workout and as you progress through each week of pregnancy*.*”*

Participant 9: “Surveys makes me mindful about my activity but not diet as I am already mindful about my diet as a vegetarian with nutritional restrictions.”

Participant 5: *“Both the app (Oura) data and REDcap questions kind of make me reflect on how I’ve been handling my emotions*, *which makes me identify any negative emotions when they happen and helps me deal with them better*. *Same with food intake*.*”*

II. Reminding potential needs: Some participants reported the benefit of the survey questions in ring (30% (n = 5/15)) and survey questions (46% (n = 7/15)) in reminding the potential behavioral and lifestyle accommodations.

Participant 8: *“I can tell what I’ve eaten and what is missing in my diet*. *So*, *eventually*, *I included the missing nutrition into my meals*. *Like*, *I noticed I was not eating enough legumes*.*”*

Participant 9: *“Sometimes it (Oura app) shows my O2 saturation under 98%*. *I have noticed this happens when I am laying down more than usual*. *So*, *I try to stay up more to avoid this*.*”*

## Discussion

The remote and smart monitoring systems using smart devices and platforms offer significant physical, mental, and economic advantages when complemented to the traditional prenatal care. This shift is particularly beneficial for pregnant individuals, providing them with reduced stress, cost-effectiveness, and the potential to prevent adverse pregnancy outcomes. In our study, we investigated the feasibility of smart remote monitoring during the second and third trimesters, along with one week postpartum. Our findings indicated that adherence to the smart platform is influenced by various factors including the gestational week, frequency of surveys, safety, and required motherhood adjustments. Findings demonstrated that the survey completion rate and Oura ring wearing time length often remained higher than 80% throughout pregnancy and gradually, with fluctuations, decreased toward the end of the study suggesting a reduction in adherence toward the end of pregnancy, with the postpartum phase exhibiting the lowest average values for both survey completion and wearing length.

The study finding of Sarhaddi et al [31] supported our findings suggesting that the smart device wearing time decreases during pregnancy and postpartum, although their average wearing length was lower compared to our study. This variance could be attributed to Sarhaddi’s study participants who were Finnish pregnant individuals with complications which may impede device usage. Additionally, the difference in average wearing time may be influenced by the type and features of smart device used; Sarhaddi’s study utilized Samsung watch, which could affect adherence due to its larger size, different wearing area, less comfort and flexibility as compared to the Oura ring used in the current study.

Grym et al [32] findings corroborate ours in terms of adherence trend during the pregnancy course as well as the identified common barriers to ring use in postpartum, such as handling newborns, concerns about scratching the newborns, and forgetfulness. However, the adherence rate was lower than our study and matched with the studies that had used watch, the similar device type [32–33]. This may suggest the impact of device characteristics on employed a wristband for monitoring across pregnancy. In contrast to our study, they observed no consistent usage pattern throughout pregnancy and reported technical issues in 17% of participants, unlike our study.

Our study revealed a high enrollment rate of over 71% and a minimal attrition rate of less than 7% throughout the study duration, with only one participant excluded due to a broken hand. This underscores the strong acceptability of participants towards engaging in a study utilizing smart monitoring platforms during pregnancy. These findings align with those of Mendez et al [34], who reported an enrollment rate of 87% in a similar study utilizing smart monitoring platforms during pregnancy.

In a different study [35], the enrollment rate was lower (56%) than our study. This discrepancy in enrollment rates could be attributed to differences in the study populations. In this study, approximately 75% of the population identified as white with complex pregnancies, such as maternal anxiety, depression, and obesity.

In the qualitative assessment of feasibility, personal desire, social acceptability and desire, support system for health awareness, and conditions associated with device/platform efficiency. These themes were further broken down into corresponding subthemes to provide deeper insights. Notably, our study addressed the theme of "social acceptability and desire" which has not been extensively explored in previous literature. The majority of participants highlighted the significance of how their device/platform usage might influence the perceptions of others, including family, friends, and colleagues. The social acceptability domain encompassing three subdomains of social support, social stigma, and appearance suggested that individuals place value on the appearance of the device and how it is perceived by others. While there is limited research addressing social acceptability and desire as implementation factors in pregnant women within healthcare settings, existing studies have highlighted the importance of appearance in acceptance and decision-making regarding platform use [36–37]. Additionally, social support has consistently been recognized as a factor that promotes adherence to smart device usage and fosters companionship [38–39].

While stressing the benefits of smart health monitoring systems including real-time self-awareness and empowerment as extracted themes in our study, it’s important to understand their role in complementing standard care practices, especially with the current absence of clear integration guidelines. Our study emphasizes that digital solutions should be seen as complementary, not replacements, to conventional pregnancy check-ups, enhancing prenatal care efficiency. Further research and standardization are needed to effectively integrate digital solutions into prenatal care protocols and determine the extent they can be complemented.

Participants in our study valued various aspects of device/platform efficiency, including time-saving, cost-saving, and the production of accurate, personalized, real-time information, efficient administration, that align with the findings in the existing evidence [40–41]. In alignment with our study, Grua et al [42] found that personalization and self-adaptation techniques in e-Health mobile apps positively influence patients’ perceptions of these apps, indicating the desire having access to the personalized data tailored to their unique needs and preferences over general information.

Our study revealed that the majority of participants emphasized the importance of device accuracy in reflecting data, consistent with literature suggesting its critical role in enabling prompt and accurate information transfer, thereby enhancing decision-making processes[43]. Moreover, all participants demonstrated the significance of ease of device use for adherence. This aligns with findings by O’Connor et al [44], indicating that straightforward information access in digital health positively impacts patient adherence, fostering a sense of accomplishment and favorable attitudes toward technology use.

### Limitations and strengths

This study placed emphasis on the healthy minority group, Hispanic population, who face a higher risk of pregnancy complications and were underserved using existing screening tools. By addressing the implementational barriers, remote health monitoring can be optimized for early detection of any problematic symptoms or signs during pregnancy for timely interventions. Additionally, the study implemented rigorous inclusion and exclusion criteria, enhancing its internal validity. Also, given that over 80% of pregnancies fall under the category of low risk, the study’s results hold potential for broader applicability, thereby enhancing its external validity. However, the limited sample size in the study hinders its ability to be generalized to a wider population.

Despite efforts to minimize limitations, a few shortcomings were encountered in this study. The identified themes of "data protection" and "financial interest," as seen in existing literature, were not found in the current study. This may be attributed to the participant-directed nature of the chosen one-on-one interview method. Future studies in this population are encouraged to address these important factors by including relevant questions.

Furthermore, it is crucial to not that those participants who firmly declined participation exhibited a strong conviction in their decision, demonstrating little willingness to engage in negotiation or consider accommodations for participation. This firm attitude towards rejection behavior may suggest potential concerns regarding trust and privacy, emphasizing the necessity for future research efforts to carefully address these issues.

### Conclusions

This study aimed to enhance the acceptability and adherence of a wearable smart device through relational content analysis and evidence-informed strategies. Quantitative analysis revealed that adherence was influenced by gestational week and survey interval, with wearing time decreasing towards the end of pregnancy and postpartum. Despite this trend, the enrollment rate was notably high compared to similar studies, indicating strong acceptability of the smart monitoring platform among pregnant individuals.

Qualitative assessment added valuable insights to the literature by delving into the feasibility and key factors associated with digital health device use during pregnancy. The study highlighted the significance of personal desire, social acceptance, support systems for health awareness, and device/platform efficiency in promoting patient engagement and acceptance of digital health monitoring. These findings offer guidance for the development and implementation of future digital health systems, ultimately enhancing healthcare outcomes and patient experiences during pregnancy.

### Implications

The study underscores the feasibility and acceptability of smart monitoring platforms for prenatal health monitoring, with high enrollment and low attrition rates. This highlights their potential as remote monitoring tools, reducing the need for frequent in-person visits, especially beneficial for individuals with limited healthcare access or in remote areas.

Moreover, postpartum adherence emerges as a challenge, with decreased survey completion and device wearing time requiring strategies to improve postpartum adherence, such as device design, notifications, and incentives.

From individuals’ perspective, the findings carry significant implications for digital health device development and implementation. Social acceptability should be a primary consideration, addressing perceptions of significant others, family, and friends to promote user acceptance. Also enhancing device features to deliver accurate, personalized, and straightforward health information can boost patient engagement and satisfaction eventually improving digital literacy and patient health outcomes.

By addressing these implications, healthcare providers and policymakers can optimize the effectiveness and acceptance of digital health technologies, ultimately improving patient outcomes and healthcare experiences.

## Supporting information

S1 Appendix Criteria for healthy pregnancy in American College of Obstetricians and Gynecologists. (DOCX)
