# Supplementary material for: Feasibility of continuous smart health monitoring in pregnant population: A mixed-method approach
Source: PLOS Digit Health. 2024 Jun 5;3(6):e0000517. doi: 10.1371/journal.pdig.0000517 (PMC11152270; doi:10.1371/journal.pdig.0000517)
Supplement: S1 Appendix — (DOCX) [file pdig.0000517.s001.docx]

| **I.Social/personal** | **II.Obstetric history** | **III.Medical and family history** | **IV.Present pregnancy** |
| --- | --- | --- | --- |
| Age maternal (<17, >35 years) | Gravida (1 or >5) | Diabetes | Pre-pregnancy BMI ≤ 25 |
| Education (<6 years) | Para (0 or >5) | Hypertension | Height <145cm |
| Marital status (non-married) | Abortions (>2) | RENAL DISEASES | PREGNANCY-INDUCED HYPERTENSION |
| Economic status (poor) | Miscarriages (1+) | CARDIOVASCULAR DISEASES (e,g., PHLEBITS) | BLEEDING |
| Smoking, alcohol consumer, or drug user | Fetal deaths (≥1) |  | PRE-ELAMPSIA |
| Domestic violence | Bleeding in T3 | BLOOD DISEASES | Multiple pregnancy |
|  | Stillbirths (≥1) | ENDOCRINE DISEASES | ABNORMAL FETAL PRESENTATION |
|  | Previous caesarean (≥1) | NO MEDICATION USE except for perinatal vitamins and folate | GESTATIONAL DIABETES MELLITUS |
|  | Preterm deliveries (≥1) | STDs | PLACENTA PREVIA |
|  | Birth weights (<2500gr) | RESPIRATORY DISEASES | Cervix insufficiency |
|  | Infant deaths (≥1) | CENTRAL NERVOUS DISEASES | A Rh-negative woman with Rh-positive husband that not willing to take RhoGAM |
|  | Toxemia (≥1) | ABDOMINAL VICERAL DISORDERS |  |
|  | Birth defects (≥1) | GENETIC OR CONGENITAL DISORDER |  |
|  |  | AUTHOIMMUNE DISEASE |  |
|  |  | INFECTIOUS DISEASES |  |
|  |  | MENTAL ILLNESS (MENTAL DISTRESS SUCH AS STRESS, ANXIETY, AND DEPRESSON)* |  |

S1 Appendix. Criteria for healthy pregnancy in American College of Obstetricians and Gynecologists
